# Phage display-derived inhibitor of the essential cell wall biosynthesis enzyme MurF

**DOI:** 10.1186/1471-2091-9-33

**Published:** 2008-12-19

**Authors:** Catherine Paradis-Bleau, Adrian Lloyd, François Sanschagrin, Tom Clarke, Ann Blewett, Timothy DH Bugg, Roger C Levesque

**Affiliations:** 1 Département de Biologie Médicale, Université Laval, Sainte-Foy, Québec G1K 7P4 , Canada; 2 Department of chemistry, University of Warwick, Coventry, CV4 7AL, United Kingdom

## Abstract

**Background:**

To develop antibacterial agents having novel modes of action against bacterial cell wall biosynthesis, we targeted the essential MurF enzyme of the antibiotic resistant pathogen *Pseudomonas aeruginosa*. MurF catalyzes the formation of a peptide bond between D-Alanyl-D-Alanine (D-Ala-D-Ala) and the cell wall precursor uridine 5'-diphosphoryl N-acetylmuramoyl-L-alanyl-D-glutamyl-meso-diaminopimelic acid (UDP-MurNAc-Ala-Glu-meso-A2pm) with the concomitant hydrolysis of ATP to ADP and inorganic phosphate, yielding UDP-*N*-acetylmuramyl-pentapeptide. As MurF acts on a dipeptide, we exploited a phage display approach to identify peptide ligands having high binding affinities for the enzyme.

**Results:**

Screening of a phage display 12-mer library using purified *P. aeruginosa *MurF yielded to the identification of the MurFp1 peptide. The MurF substrate UDP-MurNAc-Ala-Glumeso-A2pm was synthesized and used to develop a sensitive spectrophotometric assay to quantify MurF kinetics and inhibition. MurFp1 acted as a weak, time-dependent inhibitor of MurF activity but was a potent inhibitor when MurF was pre-incubated with UDP-MurNAc-Ala-Glu-meso-A2pm or ATP. In contrast, adding the substrate D-Ala-D-Ala during the pre-incubation nullified the inhibition. The IC_50 _value of MurFp1 was evaluated at 250 μM, and the *K*_i _was established at 420 μM with respect to the mixed type of inhibition against D-Ala-D-Ala.

**Conclusion:**

MurFp1 exerts its inhibitory action by interfering with the utilization of D-Ala-D-Ala by the MurF amide ligase enzyme. We propose that MurFp1 exploits UDP-MurNAc-Ala-Glu-meso-A2pm-induced structural changes for better interaction with the enzyme. We present the first peptide inhibitor of MurF, an enzyme that should be exploited as a target for antimicrobial drug development.

## Background

**Figure 1:**
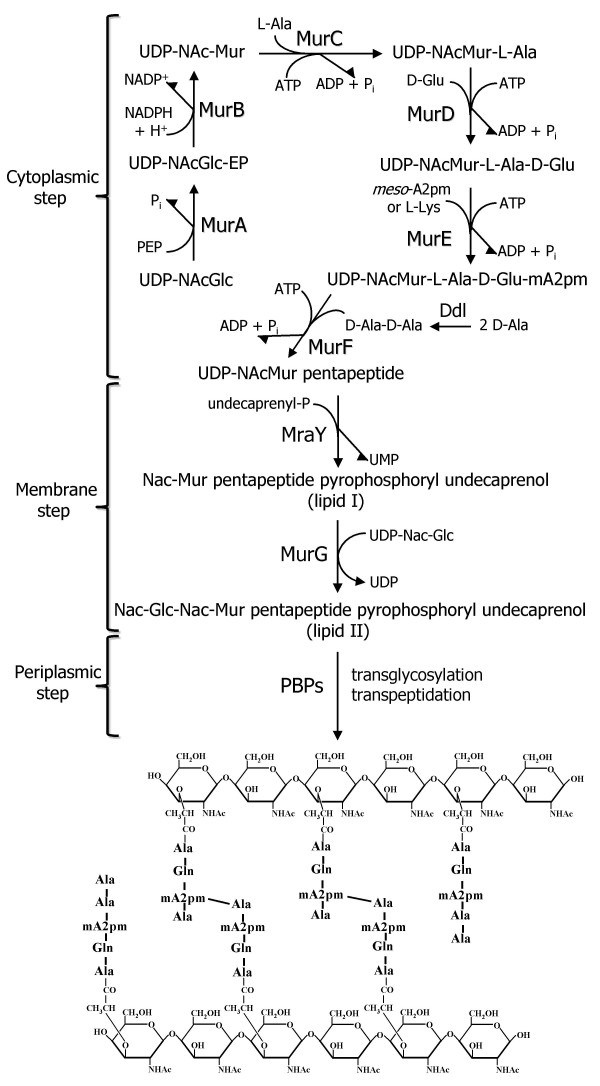
**Schematic representation of the bacterial cell wall biosynthesis pathway**. The cytoplasmic, membrane and periplasmic steps of the pathway are shown, along with the structure of the cell wall layer product, composed of alternating units of UDP-*N*-acetylglucosamine and UDP-*N*-acetylmuramic acid cross-linked via short peptide chains.

The bacterial cell wall biosynthesis pathway represents the most validated source of antibacterial targets. The pathway encodes essential and highly conserved enzymes with no eukaryotic counterparts, the inhibition of which leading to bacterial cell death [1]. The first step of the pathway is catalyzed by the cytoplasmic enzymes MurA through MurF, which synthesize UDP-*N*-acetylmuramyl-pentapeptide (Figure 1). Membrane translocases MraY and MurG then add the undecaprenyl-phosphate lipid carrier and *N*-acetylglucosamine to form lipid II. This precursor is translocated to the periplasm and linked to the growing cell wall polymer by the transglycosylation and transpeptidation actions of penicillin-binding proteins (PBPs). The cell wall layer, composed of alternating units of UDP-*N*-acetylglucosamine and UDP-*N*-acetylmuramic acid cross-linked via short peptide chains (Figure 1), maintains cell shape and integrity [2].

While several clinically useful antibiotics interfere with this pathway [3,4], no antibacterial agents target the ATP-dependent Mur ligase enzymes (MurC, MurD, MurE and MurF) that perform the non-ribosomal stepwise addition of the five amino acids forming the cell wall peptide moiety (Figure 1). This under-exploitation may be partially explained by the lack of commercially available nucleotide substrates for studying these enzymes [3,5].

We selected MurF as a specific target. MurF catalyzes the formation of a peptide bond between D-Ala-D-Ala and the nucleotide precursor UDP-*N*-acetylmuramoyl-L-alanyl-D-glutamyl-meso-diaminopimelic acid (UDP-MurNAc-Ala-Glu-meso-A2pm) with the concomitant hydrolysis of ATP to ADP and inorganic phosphate, yielding UDP-*N*-acetylmuramoyl-L-alanyl-D-glutamyl-meso-diaminopimelyl-D-alanyl-Dalanine [6]. While the roles of MurC, MurD and MurE may be substituted in a single step by the muropeptide ligase Mpl involved in cell wall recycling [7], MurF remains the sole D-Ala-D-Ala adding enzyme [8,9]. The MurF active site is highly conserved among all medically relevant bacteria [10]. Strict limitation to D-amino acid substrates [5] also makes MurF an especially attractive target for the development of antibacterial agents. These amino acids are metabolized only in prokaryotes [11], and D-Ala-D-Ala plays a critical role in cell wall cross-linking [12]. Furthermore, normally functioning MurF is essential for proper cell division, optimal expression of methicillin resistance in *Staphylococcus aureus *[9,13] and glycopeptide resistance mechanism [14,15].

Since MurF acts on a dipeptide to form bonds of a highly distinctive type, we investigated the possibility of inhibiting this enzyme with peptide ligands from a phage display library. Phage display screening allows the selection of peptides having specific binding affinities for a targeted protein and has proven useful for identification of various enzyme inhibitors including MurC and MurD [16-18]. The results we present herein constitute the first report of a peptide inhibitor of MurF. We focused this effort on the Gram-negative bacterium *Pseudomonas aeruginosa*, an ubiquitous opportunistic pathogen responsible for a variety of chronic nosocomial infections such as lung infection in cystic fibrosis patients [19]. *P. aeruginosa *is one of the most difficult microorganisms to combat due to high level of resistance to most antibiotics [20].

## Results

### Purification of biologically active MurF enzyme

The purified MurF protein was visualized as a single 52 kDa band on SDS-PAGE (data not shown). N-terminal sequencing of the first 15 amino acid residues confirmed its identity as *P. aeruginosa *MurF ligase [Swiss-Prot: Q9EY48, PIR: SF001562] (100% identical to the published sequence, including the initial Met). MurF biological activity was confirmed by mass spectrometric identification of the cytoplasmic cell wall precursor UDP-N-acetylmuramyl pentapeptide synthesized *in vitro *in the presence of the purified enzymes MurA, MurB, MurC, MurD and MurE [21].

### Affinity selection of MurF binding peptides

As shown in Table 1, each of the three rounds of bio-panning selected only a fraction of the phage input, which decreased from approximately 10^11 ^down to 10^7 ^plaque forming units (PFUs). The third round gave a lower phage recovery compared to the two previous rounds, indicating a strong selection of specific MurF-interacting peptide sequences at this step. Each elution strategy employed at the end of the third round gave similar phage recovery yields. Indeed, the competitive elution conditions using specific MurF substrates were as effective as the well-known glycine-HCl elution strategy.

**Table 1:** Phage titers obtained after each round of bio-panning using MurF and the 12-mer library

	Phage input	Eluted phages	Elution (%)
Round 1			
Gly-HCl	4 × 10^10^	8.2 × 10^6^	2 × 10^-2^
			
Round 2			
Gly-HCl	1.5 × 10^11^	4.1 × 10^7^	2.7 × 10^-2^
			
Round 3			
Gly-HCl	2 × 10^13^	1.1 × 10^7^	5.5 × 10^-5^
ATP		1.3 × 10^7^	6.5 × 10^-5^
D-Ala-D-Ala		5.6 × 10^6^	2.8 × 10^-5^

**Figure 2:**
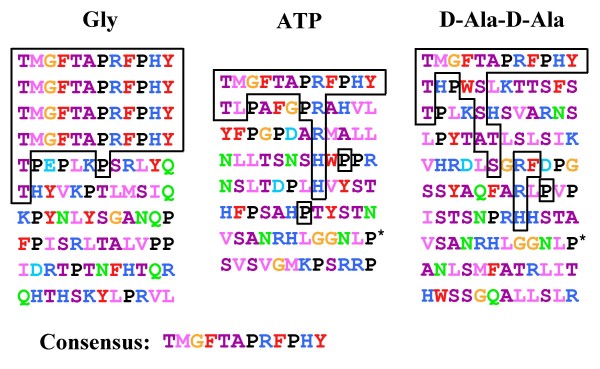
**Peptide sequences selected after the third round of bio-panning against MurF**. Acidic amino acids (D, E) are in turquoise blue, polar amino acids (Q, N) are in light green, basic amino acids (K, R, H) are in blue, hydrophobic amino acids (I, L, M, V) are in pink, hydrophobic aromatic amino acids (F, Y, W) are in red, small amino acids (A, S, C, T) are in magenta, G is in orange, and P is in black (classified according to the Venn diagram for defining the relationships between amino acids). The MurFp1 consensus sequence and related conserved motifs are boxed in black, and the star indicates the other peptide sequence recovered more than once.

The different elution conditions allowed the selection of different peptide sequences with specific motifs (Figure 2). Analysis of the frequency of peptide sequence recovery and conserved motifs identified the consensus sequence TMGFTAPRFPHY, called MurFp1. This peptide sequence was principally selected by the glycine-HCl elution strategy and was also recovered by competitive elutions (Figure 2). MurFp1 was rich in hydrophobic aromatic residues, P residues and basic residues but contained no acidic or polar residues. The N-terminal T residue and the basic R residue of MurFp1 were particularly conserved, especially in the ATP and D-Ala-D-Ala elution groups. Two hydrophobic aromatic residues or two P residues often occurred close to each other in the selected peptide sequences as they do in the consensus sequence. P residues occurred mostly near a hydrophobic aromatic residue or a basic residue, as in MurFp1 sequence (Figure 2). Apart for MurFp1 found in more than one elution group, the sequence VSANRHLGGNLP indicated by a star in Figure 2 was recovered once by both the ATP and D-Ala-D-Ala elutions. Elution with glycine-HCl gave two sequences with a "SRL" motif, which was absent in the other elution groups. Among the ATP elution group, two sequences presented a "YST" motif also absent in the other elution groups. The vast majority of the sequences eluted with D-Ala-D-Ala presented hydrophobic residues near small residues. The D-Ala-D-Ala elution group recovered more small amino acids than the other groups. Overall, the competitive elutions yielded to more sequence diversity than the glycine-HCl elution.

### Bioinformatic analysis

The theoretical pI of MurFp1 was 8.44, indicating that the peptide would be positively charged at physiological pH. MurFp1 contains a single amino acid (R) that would bear this positive charge and no amino acid bearing a counter-balancing negative charge. The ProtParam tool estimated the half-life of MurFp1 at more than 10 hours in *Escherichia coli in vivo *and at 7.2 hours in mammalian reticulocytes *in vitro*. The secondary structure prediction tools indicated with relative certainty a random coil structure. The fasts34 program did not identify any relevant protein in the UniProt database having similarities to MurFp1.

### Synthesis and purification of UDP-MurNAc-Ala-Glu-meso-A2pm

Analysis of UDP-MurNAc-Ala-Glu-meso-A2pm synthesized by the *P. aeruginosa *enzymes MurA, MurB, MurC, MurD and MurE by FPLC anionic exchange indicated a purity of 95%. The structure of the purified UDP-MurNAc-Ala-Glu-meso-A2pm product was confirmed by mass spectrometry (data not shown).

### Characterization of MurF activity

The controls performed without MurF or without any one of the substrates did not yield any phosphate from ATP (data not shown), indicating that the reaction mixture was devoid of contaminating ATPase activities that might interfere with the assay. The optimal ATP concentration was 1 mM and MurF reached noticeable activity with 100 μM of UDP-MurNAc-Ala-Glu-meso-A2pm (data not shown). Steady state kinetic analysis indicated that MurF obeyed Michaelis-Menten kinetics, reaching a maximal specific activity of 4.4 ± 0.4 μmole/min/mg at 5 mM D-Ala-D-Ala with a *K*_m _of 115 ± 10 μM (Table 2). Addition of NaCl to the reaction buffer improved MurF activity by a factor of 1.75 (data not shown). The enzyme was very sensitive to the buffer pH, with maximal activity at pH 8.6. The *k*_cat _value indicated that each MurF active site performed about 100 ligations of D-Ala-D-Ala to UDP-MurNAc-Ala-Glu-meso-A2pm and hydrolyzed about 100 ATP molecules per min, releasing the same amount of ADP and inorganic phosphate in accordance with the known stoichiometric relationship (Table 2) [22].

**Table 2:** Kinetic characterization of the MurF enzyme velocities in respect to the D-Ala-D-Ala substrate

Kinetic parameters	MurF
Specific activity (μmol/min/mg)	4.4 ± 0.1
*k*_cat _(min^-1^)	100 ± 15
*K*_m _(μM)	115 ± 10
*k*_cat_/*K*_m _(min^-1 ^μM^-1^)	0.85 ± 0.15

### Inhibitory kinetics of MurFp1

**Figure 3:**
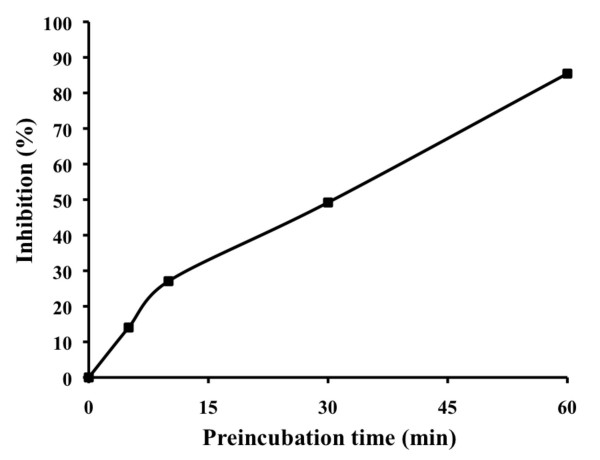
**Inhibition of MurF activity by MurFp1 as a function of pre-incubation time**. Experiments were done following different pre-incubation times of MurF with 2 mM of MurFp1.

MurFp1 was first shown to be a weak time-dependent inhibitor of MurF. The inhibition increased as a function of pre-incubation time, following an overall linear relationship. At a concentration of 2 mM, MurFp1 inhibited 50% of MurF activity subsequent to a 30-minute pre-incubation time (Figure 3).

**Figure 4:**
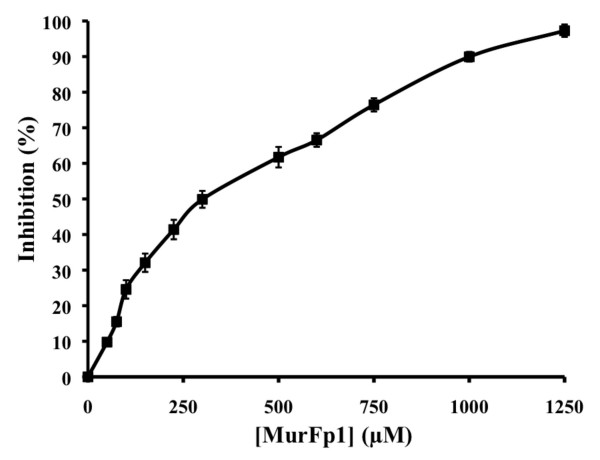
**IC_50 _determination for the inhibition of MurF activity by MurFp1**. Experiments were done following a 30 min of pre-incubation step with MurF, MurFp1 and UDP-MurNAc-Ala-Glu-A2pm.

Adding UDP-MurNAc-Ala-Glu-meso-A2pm or ATP during the pre-incubation step unexpectedly increased the inhibitory action of MurFp1, an effect less pronounced for ATP (data not shown). MurF reaction velocity in the absence of inhibitor was similar with or without UDP-MurNAc-Ala-Glu-meso-A2pm or ATP in the pre-incubation step. MurFp1 inhibited MurF eight times more efficiently when UDP-MurNAc-Ala-Glu-A2pm was added to the 30-minute pre-incubation step, giving an IC_50 _value of 250 ± 10 μM. The inhibition curve displayed a linear dose-response trend having variable slopes as shown in Figure 4.

**Figure 5:**
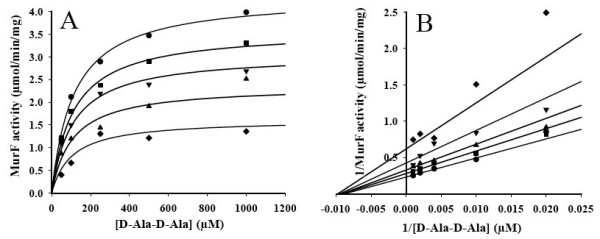
**Kinetics of MurF inhibition by MurFp1 with respect to D-Ala-D-Ala**. **A**) Michaelis-Menten and **B**) Lineweaver-Burk plots for MurF activity with respect to the D-Ala-D-Ala substrate showing inhibition by MurFp1 at 0 μM (●), 75 μM (■), 150 μM (▲), 300 μM (▼) and 600 μM (◆).

The presence of D-Ala-D-Ala during the pre-incubation step nullified the inhibition of MurF by MurFp1 (data not shown). MurF reaction velocity in the absence of inhibitor was nearly identical with or without D-Ala-D-Ala in the pre-incubation step. The *K*_i _of MurFp1 was determined with respect to the D-Ala-D-Ala substrate, with a 30 min pre-incubation of MurF with UDP-MurNAc-Ala-Glu-A2pm and MurFp1. The *K*_m _values of MurF for D-Ala-D-Ala remained almost the same for each MurFp1 concentration used to determine the *K*_i _while the *V*_max _of MurF decreased significantly as a function of MurFp1 concentration (Figure 5A and Table 3). At 600 μM MurFp1, the *V*_max _of MurF was three times lower than for the uninhibited reaction (Table 3). The slopes and the y-intercept of the fitted lines of the Lineweaver-Burk plot varied with MurFp1 concentration (Figure 5B), indicating a catalytic and specific component in the inhibition. The common intersection point of the fitted lines of the Lineweaver-Burk plot was positioned at the left of the ordinate and below the abscissa (Figure 5B), indicating a reversible mixed type of inhibition against D-Ala-D-Ala. This inhibition type was further identified as the most suitable model of inhibition, based on statistical convergence to the experimental data according to the Runs Test, giving the highest R^2 ^value (0.97), the lowest Akaike value (-275) and the lowest standard deviation of the residuals (0.15). The non-competitive type of inhibition was the second best model, giving statistical values close to the mixed model of inhibition (data not shown). The *K*_i _value of MurFp1 was 420 ± 100 μM for the mixed type of inhibition against D-Ala-D-Ala, and 370 ± 55 μM for the non-competitive model. The a parameter indicated that MurFp1 binding to the MurF-D-Ala-D-Ala complex (ES) did not significantly change MurF affinity for D-Ala-D-Ala. The β parameter indicated that the rate constant of ES breaking down to enzyme and product dropped when MurFp1 was bound to ES (Table 3).

**Table 3:** Kinetic characterization of MurF inhibition by MurFp1 with respect to D-Ala-D-Ala.

Kinetic parameters	[MurFp1] (μM)	Value
IC_50 _(μM)		250 ± 10
		
*V*_max _(nmol/min)	0	0.61 ± 0.01
	75	0.49 ± 0.01
	150	0.4 ± 0.01
	300	0.36 ± 0.02
	600	0.22 ± 0.01
		
*K*_m _(μM)	0	115 ± 10
	75	100 ± 10
	150	85 ± 5
	300	140 ± 30
	600	115 ± 25
		
Mixed α		0.85 ± 0.25
Mixed β		8.5 × 10^-10 ^± 0.6 × 10^-10^
Mixed *K*_i _(μM)		420 ± 100
		
Non-competitive β		8 × 10^-10 ^± 0.7 × 10^-10^
Non-competitive *K*_i _(μM)		370 ± 55

Kinetic parameters were monitored with a pre-incubation of 30 min with MurF, MurFp1 and UDP-MurNac-Ala-Glu-meso-A2pm.

A non-specific 12-mer peptide pre-incubated for 30 min with MurF with or without UDP-MurNAc-Ala-Glu-A2pm did not inhibit MurF activity (data not shown). The reactions were performed in the presence of excess bovine serum albumin, suggesting that MurFp1 did not interact non-specifically with proteins.

## Discussion

Phage display screening of MurF yielded to the identification of the peptide inhibitor MurFp1. The reduction of the contact time between the phages and MurF during the third round of bio-panning allowed the selection of specific MurF-interacting peptides. This selection pattern was also observed for phage display screening of the amide ligase MurD [18]. In contrast, selection of specific MurC-interacting peptides occurred earlier in the phage display screening [17]. Phage display screenings of *P. aeruginosa *proteins FtsA, FtsZ, MurC, MurD and MurF done under similar conditions did not identify any redundant sequences [17,18,23,24].

The ATPase activity of MurF has been shown to depend on the presence of both D-Ala-D-Ala and UDP-MurNAc-Ala-Glu-A2pm [22]. We developed a simple, rapid, sensitive, reliable and inexpensive assay to quantify MurF ATPase activity using the Lanzetta reagent. In opposite to other methods used to measure MurF enzymatic activity [6,8,14,22,25-36], our assay does not involve any radioactive isotope or additional enzyme, making high-throughput screening for MurF inhibitors more suitable. The maximal specific activity measured with this assay (4.4 μmole/min/mg) is comparable to the published value for the *P. aeruginosa *MurF enzyme (3.41 μmole/min/mg) [34]. The *K*_m _value determined for D-Ala-D-Ala with *P. aeruginosa *MurF (115 μM) is in the same range as values published previously for this enzyme and for its *E. coli*, *Bacillus *and *Streptococcus faecali*s equivalents [6,14,25-27,32,37]. This *K*_m _value is roughly equal to the concentration of the D-Ala-D-Ala intracellular pool in *E. coli*, established at 200 μM [6,29].

It has been shown that the binding order for substrates to Mur ligases begins with ATP, followed by the nucleotide substrate and ending with the amino acid [6,38]. It has been further demonstrated that the nucleotide substrate binds efficiently to MurD without ATP, inducing conformational changes in the C-terminal and N-terminal domains of the protein [39]. To explain how MurFp1 could be a stronger inhibitor following pre-incubation with UDP-MurNAc-Ala-Glu-meso-A2pm, we hypothesize that binding of the nucleotide substrate to MurF induces a structural change that optimizes binding and/or activity of MurFp1. *E. coli *MurF crystal structure indicates that UDP-MurNAc-Ala-Glu-meso-A2pm interacts mainly with the N-terminal domain, ATP binds principally to the central domain and D-Ala-D-Ala interacts at the C-terminus [40]. The N-terminal domain of MurF binds the uracyl ring of UDP but does not present the typical nucleotide-binding fold. Indeed, part of the central domain of MurF extends out towards the N-terminal domain to bind the pyrophosphate portion of the nucleotide substrate [40]. This suggests a conformational change upon UDP-MurNAc-Ala-Glu-meso-A2pm binding and could explain why ATP binding to the central domain also increased inhibition by MurFp1. To our knowledge, we have presented the first compound having Mur ligase inhibitory action that is enhanced by pre-incubation with the nucleotide substrate. However, appropriately substituted phosphinate inhibitors of ATP-dependent amide-forming enzymes depend on their phosphorylation via the ATP substrate. These ATP-dependent phosphinate analogues exhibit a transition-state time-dependent inhibition closely mimicking the tetrahedral intermediate involved in Mur ligase reactions [3].

Based on classical interpretation of our kinetic and statistical data, MurFp1 is a reversible mixed inhibitor. MurFp1 acted as a competitive inhibitor since the inhibition was reversed by addition of D-Ala-D-Ala during pre-incubation, and the slopes of fitted lines from Lineweaver-Burk plot increased as a function of MurFp1 concentration, revealing an increase in inhibitor binding strength [41,42]. Like a non-competitive inhibitor that interferes with substrate utilization without directly competing for the substrate binding site, MurFp1 inhibits MurF at high or low substrate concentrations, the *V*_max _of MurF decreasing as a function of MurFp1 concentration and the y-intercept of Lineweaver-Burk plot varying as a function of MurFp1. MurFp1 appears not to alter MurF affinity for D-Ala-D-Ala but rather inhibit breakdown of the enzyme-substrate complex to enzyme and product. Indeed, MurFp1 acted more like a non-competitive inhibitor than a competitive inhibitor. MurFp1 must bind elsewhere than at the D-Ala-D-Ala binding site. However, phage display and kinetic data indicated that D-Ala-D-Ala released MurFp1 from the MurF surface. We speculate that D-Ala-D-Ala interaction with MurF can enhance conformational changes that displace MurFp1. It should be noted that the glycine elution yielded to a better selection of MurFp1 than the D-Ala-D-Ala competitive elution. Furthermore, reversion of the inhibition is only noticeable when D-Ala-D-Ala is added to the pre-incubation step without the two remaining substrates. In a physiologic environment, such nullification of the inhibition would not be expected as MurFp1 inhibits MurF at high D-Ala-D-Ala concentrations. Since D-Ala:D-Ala ligases (DDl, see Figure 1) are highly sensitive to feed-back inhibition by their D-Ala-D-Ala product [37], the inhibitory potential of MurFp1 may be amplified in physiologic conditions.

MurFp1 could exert inhibition by sequestering D-Ala-D-Ala or interfering with MurF structural changes necessary for substrate proximity and amide bond formation [40]. It will be of primary interest to investigate the exact binding site of MurFp1 and whether it affects MurF conformation by co-crystallization of the MurF-MurFp1 complex with each individual substrate. This will yield valuable information for further directed optimization of the inhibitor by medicinal chemistry and peptidomimetism [43].

## Conclusion

As a first step into the development of novel inhibitors targeting unexploited bacterial targets, we have identified the first peptide inhibitor of the essential MurF amide ligase enzyme using a phage display approach. The nucleotide substrate UDP-MurNAc-Ala-Glu-meso-A2pm was synthesized and used to develop a sensitive MurF enzymatic assay. The 12-mer peptide MurFp1 acted as a time-dependent inhibitor of MurF activity and its inhibitory potential increased significantly when MurF was pre-incubated with UDP-MurNAc-Ala-Glu-meso-A2pm. MurFp1 presumably exploits UDP-MurNAc-Ala-Glu-meso-A2pm-induced structural changes for better interaction with the enzyme. The peptide inhibits the utilization of D-Ala-D-Ala by MurF, preventing the breakdown of the enzyme-substrate complex to enzyme and product with an IC_50 _value of 250 μM and a *K*_i _value of 420 μM. The discovery of this unique inhibitor opens the path to the rational design of a new generation of antibacterial agents inhibiting the bacterial cell wall biosynthesis.

## Methods

### MurF enzyme preparation

The MurF protein was over-expressed, purified and sequenced as previously described [21]. All reagents were from Sigma Aldrich (Oakville, Ontario, Canada) unless otherwise indicated. Phosphate in the MurF preservation buffer was removed by dialyzing 5 ml against 3 L of buffer A (25 mM Bis-Tris Propane pH 8.0, 1 mM β-mercaptoethanol, 2.5 mM MgCl_2_) three times at 4°C. Purified MurF was visualized on SDS-PAGE using a SYPRO ^® ^Orange staining (Bio-Rad Inc., Mississauga, Ontario, Canada) and the protein concentration was determined by the Bradford method (BioRad Inc., Mississauga, Ontario, Canada) using bovine serum albumin as standard. The phosphate-free MurF protein was aliquoted and preserved at -20°C with glycerol a final concentration of 10%.

### Affinity selection of phage displayed peptides against MurF

Phage display screening was performed with the PH.D.-12 phage library (New England Biolabs, Mississauga, Ontario, Canada). Peptide permutations are fused to the minor coat protein pIII of phage M13 via the flexible linker Gly-Gly-Gly-Ser. Three rounds of bio-panning were performed with increasing specificity obtained by increasing the Tween concentration during washes and decreasing the time of contact between the phages and the targeted protein as previously described [17,23]. Phages encoding selected peptides were eluted at the end of the third round using 100 μl of 0.2 M glycine-HCl (pH 2.2) [17], and 100 μl of 1 mM ATP or 1 mM D-Ala-D-Ala for 30 min. The DNA of ten phages was prepared and sequenced with a -96 gIII primer (New England Biolabs) for each elution condition [23].

### Bioinformatic analysis of the MurFp1 sequence

The consensus peptide sequence (TMGFTAPRFPHY) identified by phage display bio-panning was named MurFp1. The MurFp1 peptide sequence was characterized using different bioinformatic analysis algorithms of the ExPasy server including the Compute pI/Mw and ProtParam tools [44] as well as the PSIpred and SSpro secondary structure prediction tools [45]. The MurFp1 sequence was also analyzed using the fasts34 program from the FASTA software package, previously identified as the most efficient tool for identifying proteins with similarities to small peptide sequences [18,46].

### Synthesis and purification of UDP-MurNAc-Ala-Glu-meso-A2pm

Synthesis of the UDP-MurNAc-Ala-Glu-meso-A2pm substrate was performed as previously described [47] except that the reactions starting from UDP-*N*-acetylglucosamine were carried out in a single mixture using the combined activities of *P. aeruginosa *enzymes MurA, MurB, MurC, MurD and MurE. The product was purified from the reaction mixture by first removing the Mur enzymes by ultrafiltration. The ultrafiltrate was then purified by anion exchange chromatography at room temperature on DEAE-Sephacel pre-equilibrated in 10 mM ammonium acetate at pH 7.5, using a 10–800 mM ammonium acetate (pH 7.5) gradient for elution. UDP-MurNAc-Ala-Glu-meso-A2pm was identified by a pyruvate kinase/lactate dehydrogenase coupled assay for D-Ala-D-Ala-dependent ADP generation by MurF. Fractions thus found to contain UDP-MurNAc-Ala-Glu-meso-A2pm were freeze-dried from water three times to remove the ammonium acetate. Product purity was assessed by FPLC analytical anion exchange on MonoQ™ using a 0–0.5 M ammonium acetate gradient. Structure of the purified UDP-MurNAc-Ala-Glu-meso-A2pm was characterized using negative ion electrospray mass spectrometry, and the synthesized product was stored frozen in water.

### Characterization of MurF activity by spectrophotometric assay

The ATPase activity of *P. aeruginosa *MurF was quantified by a colorimetric assay that measures the release of nanomoles of inorganic phosphate using the Lanzetta reagent [48]. The reaction mixture (100 μl), held at room temperature, contained 40 nM of purified MurF from a fresh aliquot, 1 mM ATP, 100 μM UDP-MurNAc-Ala-Glu-meso-A2pm and 5 mM D-Ala-D-Ala in buffer C (100 mM Tris-HCl pH 8.6, 40 mM KCl, 500 mM NaCl, 1 mg/ml bovine serum albumin and 10 mM MgCl_2_) [6]. Reaction time was 15 min before adding 800 μl of the Lanzetta reagent. After an additional 5 min for colour development, 100 μl of 34% (w/v) sodium citrate was added to stop the reaction [48]. The optical density at 660 nm was immediately measured with a Cary spectrophotometer (Varian, Mississauga, Ontario, Canada). The amount of inorganic phosphate released by MurF was determined by comparison to the linear portion of a phosphate standard curve with a minimum R^2 ^value of 0.99. The phosphate content of each component in the reaction was subtracted from the total phosphate measured, negative controls were done without enzyme or substrate, and assays were done in triplicate. Different concentrations of each substrate and NaCl were tested to determine optimal conditions for the study of the activity of MurFp1.

The maximal specific activity, the catalytic constant (*k*_cat_), the Michaelis-Menten constant (*K*_m_) and the enzyme efficiency (*k*_cat_/*K*_m_) were determined for MurF with respect to D-Ala-D-Ala. Kinetic parameters were determined by nonlinear regression analysis with a square matrix of enzyme velocities following the Michaelis-Menten equations using the Enzyme Kinetics Module version 1 of SigmaPlot version 8.

### Kinetics of MurF inhibition by MurFp1

MurFp1 was synthesized and purified as previously described [17]. Peptide purity (> 95%) was analyzed by HPLC and molecular mass (1424.68 daltons) was confirmed by MALDI-TOF mass spectroscopy. The peptide was dissolved in buffer B (200 mM Tris-HCl pH 8.0) at a final concentration of 100 mM and the pH was adjusted to 7.0.

Inhibition of MurF enzymatic activity by MurFp1 was measured by the spectrophotometric assay described above. The substrates were added after 30 min of pre-incubation of MurF with MurFp1 and the specific activity of MurF was compared with and without MurFp1. Tests were also done in which UDP-MurNAc-Ala-Glu-A2pm was added for pre-incubation with MurF and MurFp1. The concentration of MurFp1 required to reach 50% inhibition (IC_50_) was determined and pre-incubations for 0, 5, 10, 30 and 60 min with 2 mM MurFp1 were tested. The effect of non-specific peptides was analysed on MurF enzymatic activity, with and without UDP-MurNAc-Ala-Glu-A2pm in the pre-incubation step.

To identify potential interactions between MurFp1 and the MurF substrates, each substrate was added for pre-incubation with MurF, with or without 2 mM MurFp1. The MurFp1 inhibitory constant (*K*_i_) was determined for D-Ala-D-Ala using reaction velocity data obtained with a 30 min pre-incubation of MurF with UDP-MurNAc-Ala-Glu-A2pm and MurFp1. Inhibitor concentrations tested were 0, 75, 150, 300 and 600 μM with D-Ala-D-Ala concentrations of 50, 100, 250, 500 and 1000 μM, using fixed optimal concentrations of the remaining substrates.

Kinetic data were fitted to the appropriate model equations using the Sigmaplot Enzyme Kinetics Module. Steady-state inhibition kinetic parameters were determined by nonlinear regression analysis using the least squares method as a reference to ensure accurate dataset fitting and to generate error estimates for each individual observation. The Michaelis-Menten plot of MurF initial velocity as a function of substrate concentration was used to determine the apparent kinetic parameters *V*_max _and *K*_m _with respect to D-Ala-D-Ala using equation 1 [41,42]:

(1) v = *V*_max _* [S]/(*K*_m _+ [S])

The type of inhibition was identified using the Lineweaver-Burk plot of reciprocal MurF initial velocity to reciprocal D-Ala-D-Ala concentration for the various MurFp1 concentrations. Competitive *K*_i _value was determined using the Dixon graphic re-plotting of reciprocal initial velocity versus MurFp1 concentration for each substrate concentration, which provides the inhibition components regardless of the inhibition type according to equation 2:

(2) v = *V*_max_*((1+β*I/(α*K_i_))/(1+I/(α*K_i_)))/(1+*K*_m_/S)*(1+I/*K*_i_)/(1+I/(α*K_i_)))

where *V*_max _is the maximum initial velocity for the uninhibited MurF reaction, I is MurFp1, *K*_m _is the Michaelis constant for the uninhibited reaction, α is the *K*_m _factor change when MurFp1 is bound to the MurF-D-Ala-D-Ala complex (ES) and β is the *K*_p _(the rate constant when ES breaks down to enzyme and product) factor change when MurFp1 is bound to ES [41,42].

The non-competitive *K*_i _was calculated using equation 3:

(3) v = *V*_max_/((1+*K*_m_/S)*(1+I/*K*_i_)/(1+I*β/*K*_i_))

## Authors' contributions

CPB performed all the described scientific manipulations with the exception of the synthesis and purification of UDP-MurNAc-Ala-Glu-meso-A2pm, achieved by AD, TC, AB and DHB. The kinetic analysis of MurF inhibition by MurFp1 was done by CPB with the help of FS. RCL conceived the study. CPB, FS and RCL oriented the study. CPB interpreted the data, prepared the figures and tables, and wrote the manuscript under the supervision of RCL. All authors read and approved the final manuscript.
